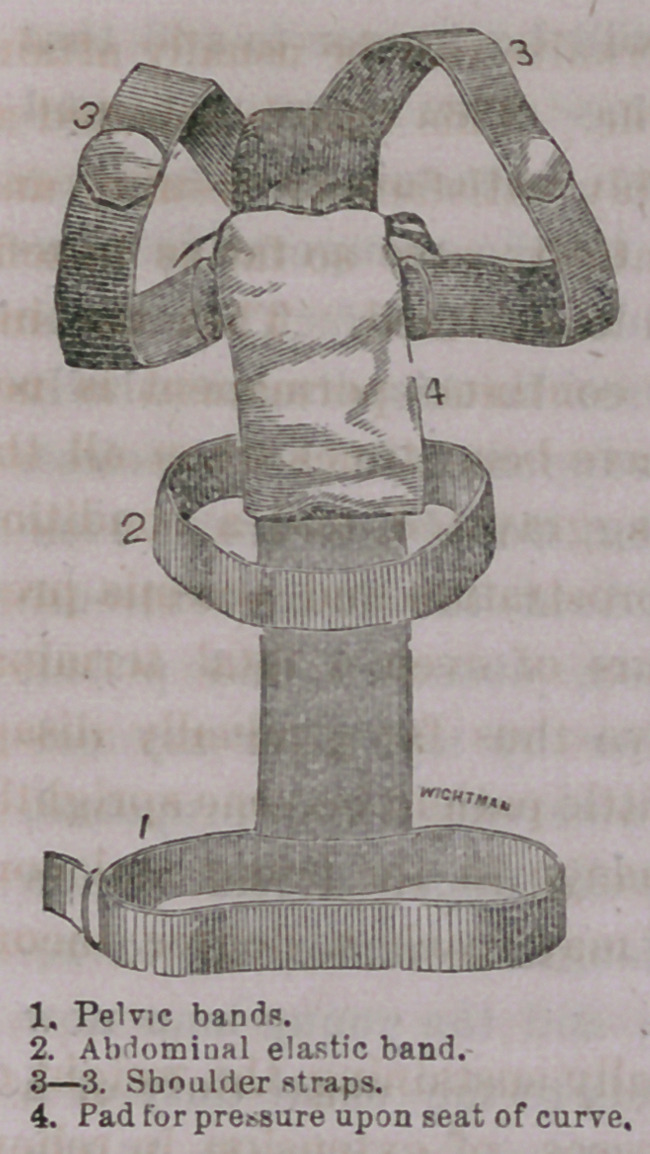# Cases in Orthopædic Surgery, with Remarks and Illustrations

**Published:** 1865-09

**Authors:** J. F. Miner


ART. III.—*Cases in Orthopædic Surgery, with remarks and illustrations.* By J. F. Miner, M. D.

Some attention to the orthopædic department of surgical practice for the past few years, has furnished cases illustrative of almost every deformity common to surgeons; to give a description of some few representative ones, their treatment and its results so far as known, will be the object, not however expecting to present anything essentially new. If any interest is attached to them, it must arise mainly from the fact, that they have been treated in private practice, without the machinery of orthopædic institutions or attendance other than the parents or friends, and with such instruments as can be improvised by most surgeons, whatever may be the field of their operations. That better results might have been obtained under more favorable circumstances is quite possible, but such as they are, it will be the object to furnish, together with a description of the cases and the means adopted for their relief or cure.

Everywhere surgeons meet with deformities which from the circumstances of the case they must treat as they best may, or wholly neglect, as is too often the case, from want of settled opinions as to what may reasonably be expected, conscious that much more is claimed by special practitioners in these diseases than they themselves are able to realize, and also from a sense of inability to efficiently treat such cases with the means at command for mechanical support, rightly regarding this as the chief dependence.

Deformities of the spine are perhaps more frequent than of any other part of the body, and constitute a condition of disease concerning which there is as great a variety of opinion among surgeons as exists concerning any one malady. It is quite useless to discuss the various theories which have been proposed as to the causes of this disease; that it may arise from a variety of influences, and is not uniformly dependent upon any one, is quite certain, though the idea that scrofula is the almost uniform cause, has gained a wide circle of supporters. The notion of "disturbed muscular antagonism," has been adopted by some and made to constitute the basis for a special system of practice. Muscular contraction has also been proposed as the chief cause of deformity. Change in the bone or cartilage, has also been regarded as the great cause of deviation in the position of the spine.

Whatever may have been suggested as the invariable cause, it appears certain that it springs from different causes and is associated with a variety of morbid conditions, all attempts to trace it to any one cause in its various forms having thus far failed. It often arises from both constitutional and local causes, and is not invariably associated with any one constitutional bias. Early diagnosis is of the first importance, since it is possible in this disease to prevent, what it is impossible to cure, though "Spine Doc tors" propose to cure almost every condition of deformity which can be conceived, while surgeons who observe the disease quite frequently, rarely expect to obtain such astonishing results. As early diagnosis is essential in preventing a disease which cannot be cured, so a careful differential diagnosis is necessary in making prognosis as to the results of treatment—in estimating the amount of relief which may be expected from the best directed efforts. Recent partial deviation of the spine can usually be restored in cases without well marked constitutional bias, and without organic change in the bones or articulating cartilages, while cases of long standing which are associated with constitutional disease, or those which have attained to great deviation and become accustomed to the unnatural position cannot safely be restored to the original form.

It has been observed that curvature arises from different causes, and is not uniformly associated with the same constitutional bias; still it may be true that curvature more generally progresses to a condition of organic change in the vertebral bones and cartilages when it occurs in scrofulous subjects. As it arises from various causes and is associated with constitutional tendencies, or may be present without such bias, so it presents a variety of therapeutical indications which might perhaps be considered in groups to some extent, but are yet almost as numerous as the cases themselves, no two presenting the same symptoms or showing themselves amenable to the same means of relief—same medical treatment, or even the same sort of mechanical support. One case will demand rest as absolute as possible for a time, while another will be benefited by exercise and air, or one will be relieved by tonics and stimulants, while another would derive no benefit from such sources.

When curvature is present and the symptoms become unmistakable, the practice is still common to attack the neighboring tissues violently and apply seatons, moxæ, leeches, blisters or actual cautery even, with the view that a derivative action will be obtained—a new inflammation set up in contiguous tissues to the relief of the cartilage or bone, whichever is supposed to be the seat of actual lesion. Clinical facts are presented to sustain the correctness of this practice, and some even of our recent standard works recommend its adoption. Without stopping to consider the grounds of objection in detail, it will be sufficient for our present purpose to say that this is rarely if ever necessary or useful, and is believed to be unphilosophical and absurd, based upon a false notion of the action of such remedies and of the true pathology of curvature of the spine. Objection, however, rests mostly upon theoretical grounds, for it may as well be confessed, that such an expedient has never been thoroughly tried, by the author and its effects watched. It is a part of the system of "counter irritation" which is gradually and fortunately disappearing from the curriculum of medical practice, or being modified to a consistancy which makes it less objectionable and injurious. Rest in horizontal position is insisted upon as the *sine qua non* of relief by many practitioners; and this position is much more tenible and rational, though this must be adopted after a great many exceptions and qualifications. In extreme cases with great tenderness and disorganization, it would perhaps be advisable, for a time, but this practice of absolute rest in the horizontal position until relief is obtained, must be modified to suit the circumstances and constitutional peculiarities of different cases.

Mechanical support is undoubtedly the chief reliance in cases which admit of its application; this, too, requires great care in its adoption, that we attempt all that is safe, and avoid a harshness which is often injurious and even dangerous. The chief indications are to relieve pressure, to restore form and afford rest, and this is to be accomplished without prejudicing the general health. To how great an extent these objects may be gained depends upon a great variety of circumstances, which all will readily appreciate.

FERDINAND HAITZ, a little boy six years old, was presented for advice in 1864; was brought to the office perfectly paralyzed, from spinal curve, which was situated in the lower cervical region. He was pale and emaciated and completely unable to use either feet or hands; upright position was painful and could not be borne for any considerable time, the weight of the head being too great for the spine to support. He had been treated for several months by various practitioners, mostly irregular, and from the spinal and sternal regions were removed two heavy iron pads, poorly covered, and every way uncomfortable as well as wholly incapable of any good. These were firmly bound to each other, and to the patient, by leather straps, thus compressing the chest, and showing how absurd and barbarous an expedient it was possible for an ignorant practitioner to invent, with no other object in view than to "do something" for a malady of which he knew nothing. The history and symptoms were of scrofulous disease of the spine, and a guarded prognosis given. At the urgent request of the parents, though everything looked unpromising, a splint was improvised, which I have since improved, but which even as first used, was productive of great benefit. Two weeks from the day the patient first called, completely incapable of standing or using the arms, he was brought to see me, or rather to show me that he could not only stand but walk a few steps unaided; this improvement continued until with the aid of the splint he could walk comfortably in the street or in the garden, which has of late been his favorite resort. He has not been uniformly well, and the condition of deformity has not been cured. He still wears the splint as made and applied at his first visit, and has never been able to leave it off, not even while in bed, has ever required the extension and support which it effects. The splint was made by using a flat, thin splint of hard wood, which had previously been used for a straight thigh splint, in dressing a fracture for a little boy. This passed up the spine, was well padded to protect the parts; upon the upper end was screwed a steel spring taken from a truss, this being fitted as well as possible, and extending over the head. The lower end was securely fastened by adhesive plaster, and the head swung from the upper spring by bandages, &c., &c. Rough and imperfect as was the appliance, the mother states that he is greatly attached to it, and as they have limited means, he still wears the splint, and it answers, perhaps, the object as well, or even better, than more expensive and pretentious appliance.

The improvement was so marked that an ambrotype was requested, showing the manner of supporting the head, which was taken a few weeks after treatment was commenced. The cut will perhaps give a better idea of the appearance of both the patient and dressing than words :

That similar results can be usually attained, is not probable. This case is selected as one of remarkably satisfactory results, and not as a representative case so far as benefit by treatment is considered. That the improvement will contiaue permanent is not certain; there have been times when all the symptoms were aggravated, and a condition of the greatest prostration and anæmia present, exciting fears of even a fatal termination. These have thus far gradually disapdeared, and the little patient become sprightly again; but a danger still exists that change in the bones and consequent pressure upon the spinal cord may reach a degree inconsistent with life.

The great improvement upon partially sustaining the weight of the head, shows the value, in proper cases, of extension in relieving the pressure upon diseased surfaces.

M. S., of a neighdoring town, is another illustrative case where sustaining the head was productive of the most decided benefit. This young lad, twelve years of age, had no marked deviation in the spine, but a tenderness in the cervical region; could not walk or play but a few minutes, before he would lay down to rest his neck. The head was turned to one side, making wry-neck, in appearance, while all sudden motion was painful. In this case extension or s upport was afforded in a more perfect manner, though upon the same general plan. Finding that similar cases were frequent, an effort to supply mechanical support resulted in the manufacture of an instrument more easily applied, more comfortable and more efficient, to which might be added the spring for sustaining the head, or this might be omitted in cases where the curvature was lower down the spine, or did not require that the head should be sustained. This was made of steel springs, fitting accurately to the spinal curve, and sustained at the base by a firm steel support passing around the pelvis, somewhat in the style of a truss. The following cut represents the body of the insument, and by it a perfect idea of its construction may be gained:

1. Pelvic bands.
2. Abdominal elastic band.
3—3. Shoulder straps.
4. Pad for pressure upon seat of curve.

This instrument can be made by mechanics who follow other trades; is stuffed so as to be comfortable, and even to afford relief to the youngest patients. It is made to fit accurately to the spine, and with it may be treated with some benefit all cases of spinal curvature, capable of mechanical relief. A pad is placed which may make pressure where required in posterior curve; lateral deviation is rectified by having an arm placed on either side as different cases may require, while an extending spring coming up over the head, will afford means of support in cases of curve or disease in the cervical bones, or in any cases where it is desirable to relieve the weight of the head. This instrument may be made useful, with its modifications and additions, in the treatment of a great variety of cases, and is to be preferred to more complicated machines in some respects; is more simple, less expensive, more comfortable to the patient, and for these reasons, if for no other, more likely to prove an available adjunct in the treatment of the various deformities of the spine.

Small children often suffer from this disease, and use of any retentive apparatus is regarded as uncomfortable, and with them, almost impossible of application; this is a mistake: it is not infrequent to obtain the greatest relief. *Extension* is often desirable, but *support* is useful; support which shall secure rest, without extending force, is oftentimes curative. As in fracture of bones, so in curvature of spine, rest will allow of cure, not, perhaps, without deformity, but, nevertheless cure, while in many cases, it is believed that to attempt perfect restoration of form, would be to insure permanent disease.

It will be observed how very imperfectly the object of this paper has been accomplished, so far, at least, as report of cases with results, good and bad, was designed; this failure is from want of space to publish, and not from change of purpose. Some good results have been reported, and it will be proper to add that failure to accomplish any great good, has often been met, and sometimes fears of having done harm even, entertained.

It would not be doing full justice to the recuperative powers of nature, should omission be made of the fact, that she oftentimes accomplishes most signal cures in this disease wholly unaided by art. A case which was supposed to be hopeless, was some years since admitted to the Buffalo General Hospital. The young man was paralyzed in the lower extremities, and nearly helpless; was examined, observed and pitied, but no attempt was made at cure, either by myself or my colleagues. Various expedients were at different times proposed, but none adopted. The curve was in the upper dorsal region and the deformity great. Nature at length commenced to repair or to adapt to existing conditions, and the young man now walks without cane or crutch, and attends to the usual duty of a grocery clerk with acceptable ability. He was an inmate of the hospital two years or more, and finally discharged himself for more lucrative employment, an illustrative example of the influence of time in effecting cures, which had treatment—mechanical support, for instance, been applied, would have been regarded as signal triumphs of art.

## Figures and Tables

**Figure f1:**
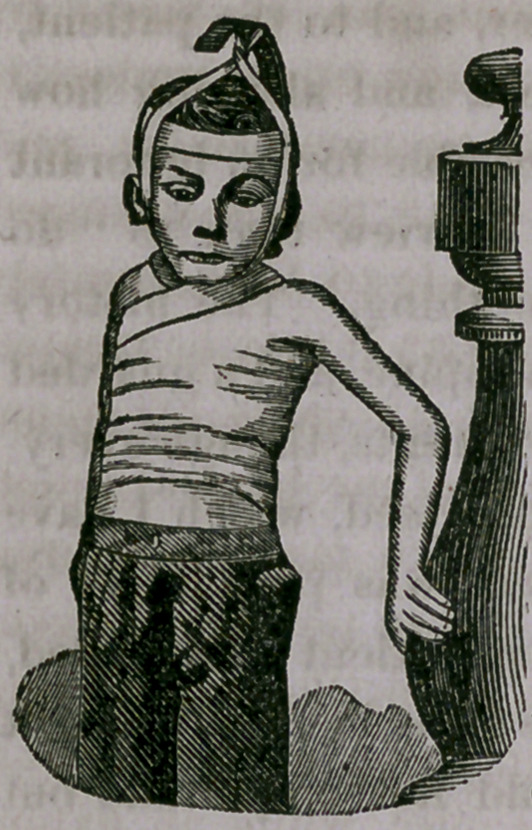


**Figure f2:**